# Imported Leishmaniasis in Dogs, US Military Bases, Japan

**DOI:** 10.3201/eid1612.100389

**Published:** 2010-12

**Authors:** Yuta Kawamura, Isao Yoshikawa, Ken Katakura

**Affiliations:** Author affiliations: Hokkaido University Graduate School of Veterinary Medicine, Sapporo, Hokkaido, Japan (Y. Kawamura, K. Katakura);; US Army Japan District Veterinary Command–Zama Branch Kangawa, Japan (I. Yoshikawa)

**Keywords:** Canine leishmaniasis, dogs, imported, Japan, Leishmania infantum, US military base, PCR, rk39, skin biopsy, letter

**To the Editor:** Leishmaniasis is found in canids in ≈50 of the 88 countries where leishmaniases are found in humans (*1*). In Japan, 2 cases of imported canine leishmaniasis have been documented in dogs from Spain (*2**,**3*). We report 2 cases of leishmaniasis in dogs in which dermatitis developed mainly on the face. Leishmaniasis was diagnosed from results of a serologic rk39 test, followed by PCR of skin lesion specimens for the *Leishmania* spp.–specific small subunit (SSU) rRNA gene. Because the dogs had lived on a US military base in Sicily, Italy, for 3 years before their owners were transfered to Japan, the animals were likely infected with *L*. *infantum* in Italy.

Animal 1 was a 6-year-old female dog that had lived in Sicily for 3 years, since 2003, and had been brought to Japan in September 2006. While she lived in Italy, she had exhibited alopecic, pruritic, and crusty skin lesions, mainly around the face and on the forearms and hind legs.

In November 2006, the dog was brought to the US Army Veterinary Command’s Zama Veterinary Treatment Facility with dermatitis (Figure A1, panel A) and additional signs of kidney failure. A serum specimen was positive by the rk39 dipstick test for diagnosis of visceral leishmaniasis (Kalazar Detect; InBios, Seattle, WA, USA). A skin punch biopsy specimen was obtained for cultures and PCR for the parasites in December 2006. Cultures of 4 skin specimens were all negative, probably because of cool transportation of the samples for 1.5 days before the cultures were started. The dog’s condition was treated with ketoconazole and then allopurinol. The skin conditions initially improved, but the lesions did not completely resolve (Figure A1, panels B–D). In May 2008, the dog was humanely killed because of central vestibular disease with unknown cause. A necropsy was not performed.

Animal 2 was a 12-year-old male dog that had also lived in Sicily for 3 years since 2000, and was brought to Yokosuka Base in Japan in 2003. In January 2004, the dog was positive for visceral leishmaniasis by the rk39 test; no particular clinical signs were observed.

In March 2007, the dog was referred to Zama Veterinary Treatment Facility with pruritic alopecia on the dorsum and head, and a skin punch biopsy specimen was obtained for histopathologic evaluation. The presence of amastigotes of *Leishmania* species within areas of dermal inflammation was confirmed at the Armed Forces Institute of Pathology (Washington, DC, USA). In April 2007, a second skin punch biopsy specimen was obtained for PCR.

PCR was performed for the *Leishmania*-specific SSU rRNA gene (*4*). For primary PCR, primers R221 (5′-GGTTCCTTTCCTGATTTACG-3′) and R332 (5′-GGCCGGTAAAGGCCGAATAG-3′) were used. For nested PCR, primers R223 (5′-TCCCATCGCAACCTCGGTT-3′) and R333 (5′-AAAGCGGGCGCGGTGCTG-3′) were used. In the primary reaction, the expected PCR products of ≈603 bp were detected in 2 of 4 skin DNA specimens from patient 1 and 1 of 5 skin DNA specimens from patient 2 (Figure, panel A, lanes 2, 3, 9). In the nested reaction, the expected PCR products of ≈359 bp were seen in all 4 specimens from patient 1 and in 4 of 5 specimens from patient 2 (Figure, panel B, lanes 1–4, and 5, 6, 8, 9); some bands were faint. The nucleotide sequences (288 bp) of the nested PCR product of patient 1 were 100% identical to those of patient 2 and sequences of the SSU rRNA gene of *L.* *infantum* (IPT1 strain, used as a positive control), *L. infantum* (M81429), *L.* *donovani* (M80295), and *L.* *chagasi* (M81430).

Global warming, which causes changes in the distribution of the sand fly vectors, and human-produced risk factors, such as travel, migration, and urbanization, may increase the incidence of leishmaniasis (*5*). Military mobility and operations are also a major risk factor for leishmaniasis in humans and canids (*6*). In Japan, of >300 kala-azar (visceral leishmaniasis) patients reported, 218 were soldiers who returned from the People’s Republic of China before and after World War II (*7*). In the present study, 2 dogs infected with *L.* *infantum* had been brought to Japan from Italy by US military families.

Dog-to-dog transmission by direct contact with contaminated blood through biting may explain the recent outbreaks of leishmaniasis in foxhounds in North America (*8*). In Japan, although no sandfly species that could transmit leishmania have been reported (*7*), direct dog-to-dog transmission of leishmaniasis can occur. *Babesia gibsoni* infection is prevalent among fighting dogs in Japan, likely because of the transmission of infected erythrocytes through biting (*9*). Greater sharing of information and of diagnostic procedures is required in Japan because few medical and veterinary practitioners have experience with leishmaniasis patients.

**Figure:**
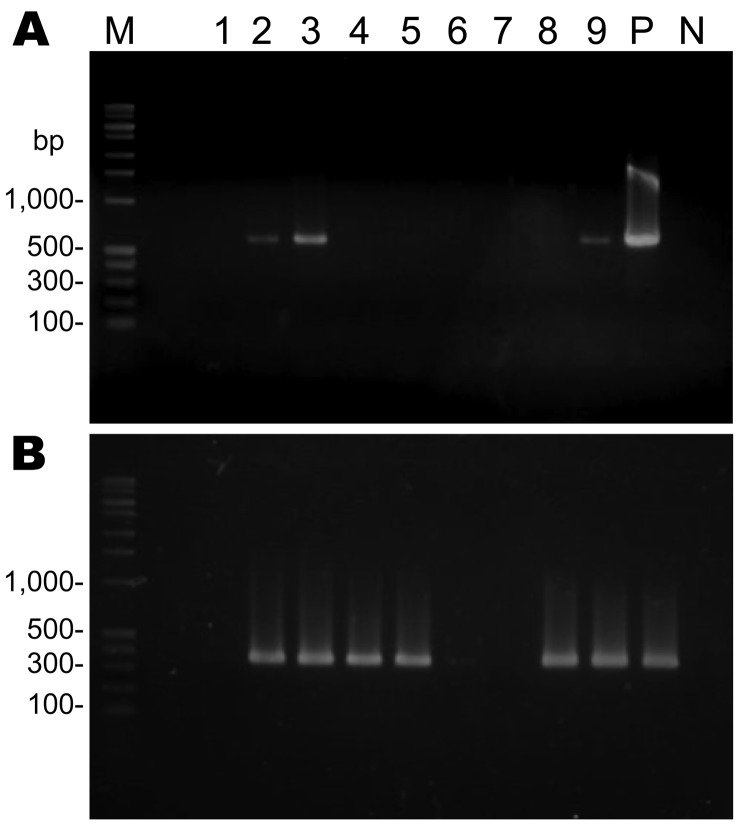
PCR amplification of the *Leishmania* spp.–specific small subunit rRNA gene from skin biopsy specimens from infected dogs, Japan. DNA samples (100–200 ng) were subjected to primary PCR (A), followed by nested PCR (B). Lanes 1–4, skin DNA samples from patient 1; lanes 5–9, skin DNA samples from patient 2; M, DNA molecular marker; P, positive control; N, negative control.

## Figures and Tables

**Figure A1:**
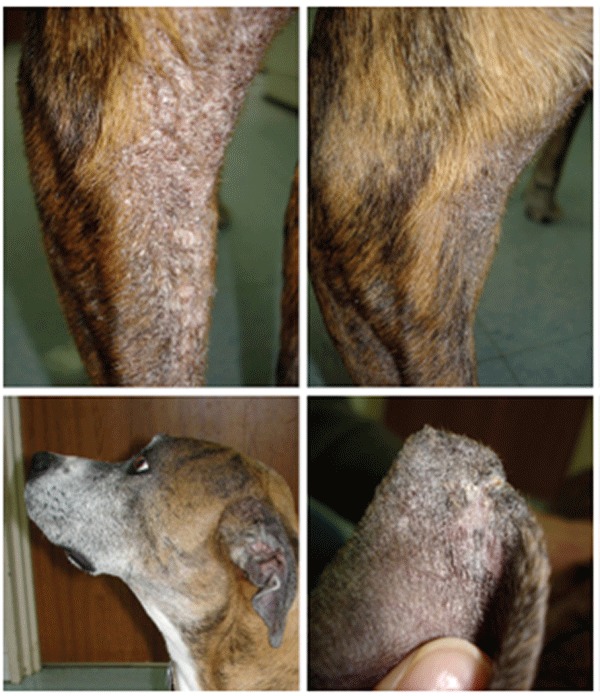
Animal 1 with alopecic, pruritic, and crusty skin lesions mainly around the face, head, margins of ear pinnae, cranial aspect of the elbows and forearms, and caudal aspect of the hind legs. The lateral aspect of the left hind leg before treatment (A) and after treatment (B) (ketoconazole and then allopurinol for 3 months). The lateral aspect of the face (C) and the inner aspect of the left ear pinna (D) after the same treatment.
